# Identification of Mg^2+^ ions next to nucleotides in cryo-EM maps using electrostatic potential maps

**DOI:** 10.1107/S2059798321001893

**Published:** 2021-03-30

**Authors:** Jimin Wang, S. Kundhavai Natchiar, Peter B. Moore, Bruno P. Klaholz

**Affiliations:** a Department of Molecular Biophysics and Biochemistry, Yale University, New Haven, CT 06520-8114, USA; b Centre for Integrative Biology (CBI), Department of Integrated Structural Biology, IGBMC, CNRS, Inserm, Université de Strasbourg, 1 Rue Laurent Fries, 67404 Illkirch, France; c Institute of Genetics and of Molecular and Cellular Biology (IGBMC), 1 Rue Laurent Fries, Illkirch, France; d Centre National de la Recherche Scientifique (CNRS), UMR 7104, Illkirch, France; e Institut National de la Santé et de la Recherche Médicale (Inserm), U964, Illkirch, France; f Université de Strasbourg, Illkirch, France; g Department of Chemistry, Yale University, New Haven, CT 06520-8107, USA

**Keywords:** cryo-EM, Mg^2+^ ions, nucleotides, electrostatic potential maps, charge effects

## Abstract

Cryo electron microscopy (cryo-EM) can produce maps of macromolecules that have resolutions that are sufficiently high that structural details such as chemical modifications, water molecules and bound metal ions can be discerned. However, those accustomed to interpreting the electron-density maps of macromolecules produced by X-ray crystallography need to be careful when assigning features such as these in cryo-EM maps because cations, for example, interact far more strongly with electrons than they do with X-rays. Using simulated electrostatic potential (ESP) maps as a tool led us to re-examine a recent cryo-EM map of the human ribosome, and we realized that some of the ESP peaks originally identified as novel groups covalently bonded to the N7, O6 or O4 atoms of several guanines, adenines or uridines, respectively, in this structure are likely to instead represent Mg^2+^ ions coordinated to these atoms, which provide only partial charge compensation compared with Mg^2+^ ions located next to phosphate groups. In addition, direct evidence is provided for a variation in the level of 2′-*O* ribose methylation of nucleotides in the human ribosome. ESP maps can thus help in identifying ions next to nucleotide bases, *i.e.* at positions that can be difficult to address in cryo-EM maps due to charge effects, which are specifically encountered in cryo-EM. This work is particularly relevant to nucleoprotein complexes and shows that it is important to consider charge effects when interpreting cryo-EM maps, thus opening possibilities for localizing charges in structures that may be relevant for enzymatic mechanisms and drug interactions.

## Abstract 2

Cryo-EM can produce maps with resolutions that are sufficiently high that fine structural details can be discerned. Using calculated electrostatic potential maps, it is shown that it is possible to identify Mg^2+^ ions bound to nucleotide bases at which no charge compensation occurs, in contrast to Mg^2+^-bound phosphate groups, in the ribosomal RNA.

## 1. Introduction   

The impact of high-resolution cryo-EM on structural biology has increased dramatically over the past few years thanks to the development of improved direct electron detectors and image-processing methods, which also includes structure sorting by classification and methods to resolve less ordered regions by focused classification and refinement (Orlov *et al.*, 2017 ▸; Chiu & Downing, 2017 ▸; Ognjenović *et al.*, 2019 ▸; von Loeffelholz *et al.*, 2017 ▸; Klaholz, 2015 ▸; Orlova & Saibil, 2010 ▸; Khoshouei *et al.*, 2017 ▸; Banerjee *et al.*, 2016 ▸; Bartesaghi *et al.*, 2015 ▸; Cheng, 2015 ▸; Nakane *et al.*, 2018 ▸; Costa *et al.*, 2017 ▸). Cryo-EM maps resemble the electron-density maps generated by X-ray crystallography, and consequently microscopists are interpreting their maps in the same way as X-ray crystallo­graphers do (Brown *et al.*, 2015 ▸; Natchiar *et al.*, 2017*a* ▸; Afonine, Poon *et al.*, 2018 ▸; Afonine, Klaholz *et al.*, 2018 ▸). While this practice may be appropriate as a means for a microscopist to obtain an initial atomic model from his or her map, it is important that at some point the difference in physical properties between cryo-EM and X-ray crystallo­graphic maps be taken into account. Cryo-EM maps are electrostatic potential (ESP) maps to which the charges of both nuclei and electrons contribute, and they are much more sensitive to atomic charges than X-ray maps, which report only on the locations of electrons (Wang & Moore, 2017 ▸; Wang *et al.*, 2017 ▸, 2018 ▸, 2020 ▸; Hryc *et al.*, 2017 ▸; Marques *et al.*, 2019 ▸; Gisriel *et al.*, 2020 ▸).

Here, we compare calculated ESP maps of Mg^2+^ ions bound to nucleotide bases with the ESP maps of several bases as visible in the cryo-EM map of the human ribosome (Natchiar *et al.*, 2017*b* ▸). This structure was recently determined to a resolution (average resolutions of 2.9, 3.0 and 3.1 Å for the 60S ribosomal subunit and the body and head parts of the 40S ribosomal subunit, respectively) at which numerous chemical modifications such as 2′-*O*-methylations or base modifications could be visualized (Natchiar *et al.*, 2017*b* ▸). While there are chemical data that support the assignment of many of the modified bases identified in this map (*i.e.* the sites belonging to classes I and II in our original publication; Natchiar *et al.*, 2017*b* ▸), some of these assignments lacked such support (*i.e.* the class III sites that required further analysis). Of particular interest in this regard are the extra features in the ESP map adjacent to the N7, O6 or O4 atoms of the guanosines, adenines or uracils, respectively, of several of the class III bases that were initially annotated as xp^4^ and xp^6^, *i.e.* yet to be identified/confirmed chemically (Natchiar *et al.*, 2017*b* ▸). To obtain a better understanding of these features, we have compared them with ESP maps that we have calculated for hydrated Mg^2+^ ions coordinately bound to these bases at the same positions.

## 2. Results and discussion   

**Figure 1:**
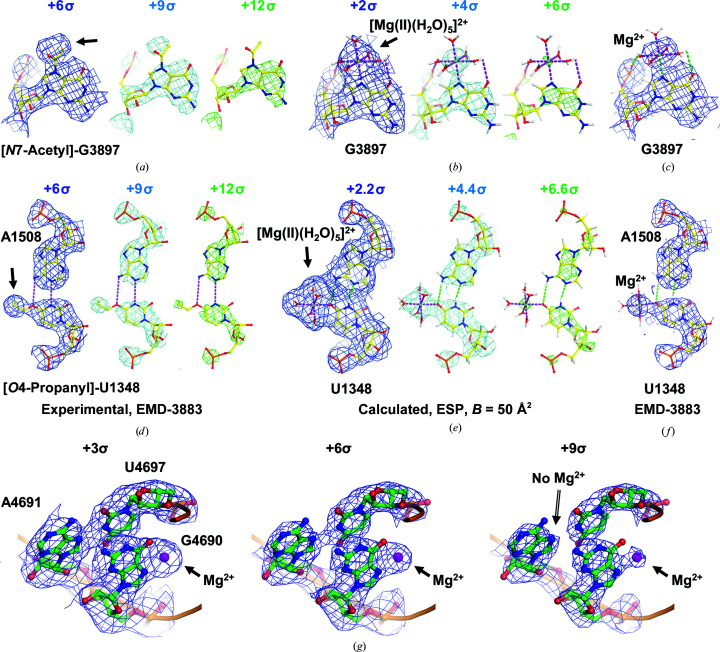
Comparison of simulated ESP and experimental cryo-EM maps for two representative residues. Experimental ESP maps contoured at three sequential levels (+6σ, blue; +12σ, cyan; +18σ, green) (*a*, *d*) and simulated ESP maps (*b*, *e*) for the previously assigned *N*7-acetyl-G3897 and *O*4-propanyl-U1348 nucleotides; (*c*, *f*) cryo-EM maps with fitted Mg^2+^. The comparison illustrates that densities close to the N7, O6 or O4 atoms of guanosines, adenines or uridines, respectively, can be misinterpreted due to the positive charge of hydrated Mg^2+^ ions that appear notably larger in cryo-EM maps compared with X-ray crystallographic maps. (*g*) Comparison of neighbouring residues with and without an Mg^2+^ ion bound; even at the higher contour level of the cryo-EM map the density remains continuous due to the positive charge that is only partially compensated by the nucleotide base and the coordinating water molecules.

To begin this analysis, we selected two residues from the class III set: G3897 and U1348 (Fig. 1 ▸). G3897 exhibits a strong and large ESP map feature adjacent to its N7 position that splits off from the ESP belonging to the rest of the base only when visualized at high contour levels (Fig. 1 ▸ *a*). This feature was originally modelled as an acetyl group covalently bonded to the N7 atom of G3897 because in maps contoured at normal levels it appeared to be continuously connected to the base. An acetyl group does fit into this density moderately well at normal contour levels, but it is clear from the simulated ESP map (Fig. 1 ▸ *b*), which was calculated as recently described (Wang *et al.*, 2018 ▸; see details in Section 2), that a hydrated Mg^2+^ ion fits it even better at all contour levels (Figs. 1 ▸ *b* and 1 ▸ *c*). Similarly, the strong ESP map feature next to the O4 atom of U1348 is better explained as a hydrated Mg^2+^ ion (Figs. 1 ▸ *d*–1 ▸ *f*). Based on these observations, we reanalyzed the density that corresponds to all of the class III nucleotides where the modifications proposed initially involved the N7, O6 or O4 atoms of guanosines, adenines or uridines, respectively. We have concluded that the extra features in the ESP maps associated with these bases represent bound hydrated Mg^2+^ ions, and we have now annotated these residues accordingly (Table 1 ▸). Our revised annotation of these nucleotides is consistent with a recent biochemical study that showed no chemical modification of these particular nucleotides in the human ribosome (Taoka *et al.*, 2018 ▸); octahedrally coordinated hydrated Mg^2+^ ions have recently also been visualized in the 50S ribosomal subunit from *Escherichia coli* (Stojković *et al.*, 2020 ▸).

**Figure 2:**
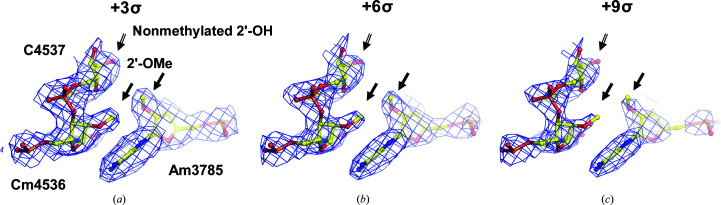
Evidence for 2′-*O*-methylation of C4536/A3785. Ribose moieties of nucleotides (2′-*O*-methyl modified Cm4536 and Am3785 as examples compared with the neighbouring nonmethylated C4537) are much less affected by partial charges (atomic model and cryo-EM map, EMBD entry EMD-3883, at three consecutive contouring levels).

The reason that the ESP map features for these Mg^2+^ ions are so large and conspicuous (see also a comparison between two nucleotides with and without Mg^2+^; Fig. 1 ▸ *g*) is that the two positive charges of the Mg^2+^ ion are only partially compensated for by the partial negative charges of the water molecule O atoms and the base N7, O6 or O4 atoms that are coordinated to them. In contrast, the full negative charges of the O atoms of the phosphate groups in the rRNA backbone are much more effective in reducing the amplitudes of the ESP peaks of any Mg^2+^ ions bound to them, which makes it easier to resolve the peak corresponding to a phosphate O atom from the peak of an associated Mg^2+^ ion. Modifications at 2′-*O* ribose positions and on the less polarized N1, N2, N3, N4 and C5 atoms of the nucleotide bases are much less affected by charge effects (Fig. 2 ▸) and give rise to ESP map features that are much easier to assign because they are so similar to the corresponding features in electron-density maps.

It is clear from the comparison of experimental and simulated ESP maps of an Mg^2+^ ion shown here that the peak of an Mg^2+^ ion in an ESP map is much larger than the corresponding peak in an electron-density map (Fig. 1 ▸). For this reason, Mg^2+^ ion peaks tend to merge with those of the neighbouring atoms in ESP maps contoured at low and normal levels (Fig. 1 ▸ *g*). This is especially true when the interaction distance is short, for example often ∼2.0–2.2 Å (Table 1 ▸), which is only slightly longer than the length of an ordinary covalent bond and is not resolvable at the resolution relevant here (hence there is some variability compared with the standard distances, which are around 2.1 Å; Dokmanić *et al.*, 2008 ▸). Peak size is less of a problem for other kinds of nucleotide modifications when there are no charged species involved.

**Figure 3:**
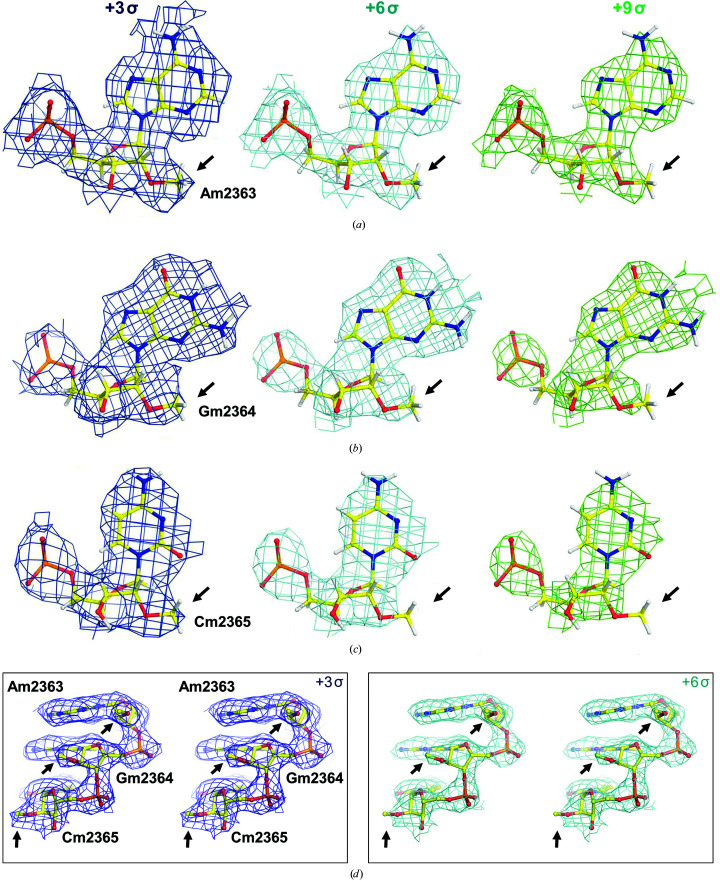
Quantitative comparison of three consecutive nucleotides with different levels of 2′-*O*-methylation. (*a*) Am2363 at three contour levels, (*b*) Gm2364, (*c*) Cm2365. (*d*) Stereo diagrams at +3σ (left) and +6σ (right) contour levels. Am2363, Gm2364 and Cm2365 have strong, medium and weak densities, respectively, suggesting differential levels of 2′-*O*-methylation.

Quantitative comparison of methylation levels at different locations in cryo-EM maps is very difficult because the resolution in one part of a map may not be the same as it is in another. Interestingly, there is one region in the ESP map of the human ribosome that contains several 2′-*O*-methylated nucleotides where the local resolution is effectively constant (Fig. 3 ▸). There it is clear that the modification levels are not the same: A2363 has the highest level of 2′-*O*-methylation, C2365 has the lowest and G2364 is in between. These observations may indicate the presence of partial modifications, *i.e.* a mixture of the absence and presence of 2′-*O*-methylation, which may be functionally relevant (Natchiar *et al.*, 2018 ▸).

In summary, our observations demonstrate how important it is to take atomic charges into account when interpreting cryo-EM, *i.e.* ESP, maps and deriving detailed atomic models (Liebschner *et al.*, 2019 ▸; Klaholz, 2019 ▸). Compared with previous studies on the localization of Mg^2+^ ions next to phosphate groups (Wang *et al.*, 2018 ▸), the novelty here is to identify ions in positions next to nucleotide bases that are particularly difficult to address in cryo-EM maps due to charge effects. These are typically attached to the N7, O6 or O4 atoms of nucleotide bases, *i.e.* at positions with only little charge compensation compared with Mg^2+^ ions located next to phosphate groups. This opens new possibilities for localizing charges in structures, which may be relevant to enzymatic mechanisms and drug interactions *etc*. This is particularly important for all structures that contain RNA or DNA in various nucleoprotein complexes, which are full of negatively charged phosphate groups, and bound counter-ions, notably divalent ions such as Mg^2+^. Until the community better understands the effects that local charges have on ESP maps, calculated ESP maps of the sort we used here may have a useful role to play when analyzing cryo-EM maps.

## 3. Methods   

Our revised annotation was made by careful comparison of the experimental ESP map with calculated ESP maps from atomic models as recently described (Wang *et al.*, 2018 ▸). For these calculations, atomic partial charges were taken from Cornell *et al.* (1995 ▸) and Pavlov *et al.* (1998 ▸), and unknown atomic *B* factors were systematically varied with an increment of 10 Å^2^. Scripts, libraries and examples are provided in the supporting information: (i) awk_NucleicAcidProtein_PDB_Kollman, which assigns charges for nucleotide and amino-acid residues according to Cornell *et al.* (1995 ▸), (ii) awk_ESP_with_charges_ions_P1_PDB, which calculates ESP structure factors with assigned charges and standard form factors for ionized atoms (Peng, 1998 ▸, 1999 ▸), (iii) HexahydratedMG_PSS1998_lib.cir, which is a crystallo­graphic information file library for a hexahydrated Mg^2+^ ion according to Pavlov *et al.* (1998 ▸), and (iv) U1348A1508Pair_HHMg_center1.pdb, which is an example for the U1348⋯A1508 base pair (see Fig. 1 ▸) with a hydrated Mg^2+^ complex included placed in a cubic *P*1 box with *a* = *b* = *c* = 30 Å.

## Supplementary Material

awk_NucleicAcidProtein_PDB_Kollman script. DOI: 10.1107/S2059798321001893/vo5001sup1.exe

awk_ESP_with_charges_ions_P1_PDB script. DOI: 10.1107/S2059798321001893/vo5001sup2.exe

HexahydratedMG_PSS1998_lib.cir library. DOI: 10.1107/S2059798321001893/vo5001sup3.txt

U1348A1508Pair_HHMg_center1.pdb. DOI: 10.1107/S2059798321001893/vo5001sup4.pdb

## Figures and Tables

**Table 1:** Reannotations to Mg^2+^ ions that have been made for densities in the vicinity of N7, O6 or O4 atoms (bold) of guanosines, adenines or uridines, respectively (28S rRNA; no changes in 18S rRNA) The observed coordination is often octahedral, which is typical of Mg^2+^, but it cannot be excluded that some positions are other ions (K^+^ is present in the buffer, but coordination around K^+^ is less regular, often with more than six ligands with longer coordination distances) or water molecules. The coordinates of the human ribosome structure in the PDB were updated accordingly. Human 28S rRNA sequence, NR_003287.2; human 80S ribosome, PDB entry 6ek0; human 80S ribosome, EMBD entry EMD-3883.

28S rRNA residue name	Previous annotation	Comments
G237	xp^6^G237	Mg^2+^ (distance between **O6** and Mg^2+^ ion is 2.6 Å)
U1348	xp^4^U1348	Mg^2+^ (distance between **O4** and Mg^2+^ ion is 2.3 Å); see also calculated ESP map (Fig. 1 ▸)
G1574	xp^6^G1574	Mg^2+^ (distance between **O6** and Mg^2+^ ion is 2.2 Å)
G1605	m^7^G1605	Weak Mg^2+^ (distance between **N7** and Mg^2+^ ion is 2.0 Å)
U1659	xp^4^U1659	Mg^2+^ (distance between **O4** and Mg^2+^ ion is 2.1 Å)
G1797	xe^7^G1797	Mg^2+^ (distance between **N7** and Mg^2+^ ion is 2.2 Å)
G1909	xp^7^G1909	Mg^2+^ (distance between **N7** and Mg^2+^ ion 2.9 Å)
G2297	xe^7^G2297	Mg^2+^ (distance between **N7** and Mg^2+^ ion 2.8 Å)
G2380	m^6^G2380	Weak Mg^2+^ (distance between **O6** and Mg^2+^ ion is 1.6 Å)
G2522	m^7^G2522	Mg^2+^ (distance between **N7** and Mg^2+^ ion is 2.8 Å)
G2754	xp^6^G2754	Mg^2+^ (distance between **O6** and Mg^2+^ ion is 2.1 Å)
G3880	xp^7^G3880	Mg^2+^ (distance between **N7** and Mg^2+^ ion is 2.7 Å)
G3897	ac^7^G3897	Mg^2+^ (distance between **N7** and Mg^2+^ ion is 2.4 Å); see also calculated ESP map (Fig. 1 ▸)
Gm3899	ac^7^Gm3899	Mg^2+^ at **N7** (distance between **N7** and Mg^2+^ ion is 2.2 Å)
G4129	m^6^G4129	Weak Mg^2+^ (distance between **O6** and Mg^2+^ ion is 1.8 Å)
G4185	m^6^G4185	Weak Mg^2+^ (distance between **O6** and Mg^2+^ ion is 2.2 Å)
U4194	xp^4^U4194	Mg^2+^ (distance between **O4** and Mg^2+^ ion is 2.2 Å)
G4355	xe^6^G4355	Mg^2+^ (distance between **O6** and Mg^2+^ ion is 2.2 Å)
G4371	m^2^xp^7^G4371	Mg^2+^ (distance between N2 and Mg^2+^ ion is 2.4 Å)
G4472	m^6^G4472	Mg^2+^ (distance between **O6** and Mg^2+^ ion is 2.1 Å)
m^6^G4529	m^6^G4529	Weak Mg^2+^ (distance between **O6** and Mg^2+^ ion is 1.7 Å)
G4550	m^7^G4550	Mg^2+^ (distance between **N7** and Mg^2+^ ion is 2.9 Å)
A4564	m^7^A4564	Possible Mg^2+^ or water molecule (distance to **N7** is 1.5 Å)
G4690	ac^7^G4690	Mg^2+^ (distance between **N7** and Mg^2+^ ion is 2.2 Å)
